# Alkaline thermal treatment of seaweed for high-purity hydrogen production with carbon capture and storage potential

**DOI:** 10.1038/s41467-020-17627-1

**Published:** 2020-07-29

**Authors:** Kang Zhang, Woo-Jae Kim, Ah-Hyung Alissa Park

**Affiliations:** 1 Lenfest Center for Sustainable Energy, Columbia University, New York, NY 10027 USA; 2 State Grid Zhejiang Electric Power Research Institute, 310014 Hangzhou, PR China; 3 Department of Chemical Engineering and Materials Science, Ewha Womans University, Seoul, 03760 Korea; 4 Department of Earth and Environmental Engineering and Department of Chemical Engineering, Columbia University, New York, NY 10027 USA

**Keywords:** Climate-change mitigation, Carbon capture and storage, Energy harvesting, Renewable energy, Chemical engineering

## Abstract

Current thermochemical methods to generate H_2_ include gasification and steam reforming of coal and natural gas, in which anthropogenic CO_2_ emission is inevitable. If biomass is used as a source of H_2_, the process can be considered carbon-neutral. Seaweeds are among the less studied types of biomass with great potential because they do not require freshwater. Unfortunately, reaction pathways to thermochemically convert salty and wet biomass into H_2_ are limited. In this study, a catalytic alkaline thermal treatment of brown seaweed is investigated to produce high purity H_2_ with substantially suppressed CO_2_ formation making the overall biomass conversion not only carbon-neutral but also potentially carbon-negative. High-purity 69.69 mmol-H_2_/(dry-ash-free)g-brown seaweed is produced with a conversion as high as 71%. The hydroxide is involved in both H_2_ production and in situ CO_2_ capture, while the Ni/ZrO_2_ catalyst enhanced the secondary H_2_ formation via steam methane reforming and water-gas shift reactions.

## Abstract (web summary)

While biomass may serve as a renewable source of carbon-neutral hydrogen, it is challenging both to utilize as-found bio-resources and to suppress CO_2_ formation. Here, authors convert wet, salty seaweed using alkaline thermal treatment to produce high-purity hydrogen and suppress carbon emission.

## Introduction

Given the rapid development of the global economy and ever-increasing population, the world’s energy demand will increase by 30% by 2040 according to the International Energy Agency (IEA)^1^. Global CO_2_ emissions from energy use are expected to increase up to 35.7 GT per year, which is far from the necessary level required to avoid severe climate change^2,3^. Thus, further use of renewable low-carbon and carbon-neutral energy sources (e.g., solar photovoltaic, wind, hydropower, and biomass) is urgently needed^4,5^.

Not all biomass can be considered sustainable resources since their cultivation, harvesting, treatment, processing, and transportation can lead to greater environmental impacts than benefits depending on the paths have been taken. For example, the transportation of low energy density biomass for long distance using fossil fuels would make biomass less attractive as sustainable resources. Nevertheless, biomass is one of the renewable resources that have a potential to be not only carbon-neutral but even be carbon-negative if combined with carbon capture and storage (CCS) scheme^6,7^. According to the U.S. Energy Information Administration (EIA), biomass contributed 5% of the U.S. primary energy supply in 2018 and is expected to replace more than 30% of U.S. petroleum consumption by 2030^8^. Most of the biomass is terrestrial. Their limited availability compared to fossil resources, the associated land use change, and the need for freshwater for cultivation and growth are often used to argue against the large-scale long-term impact of bioenergy^9,10^.

**Fig. 1:**
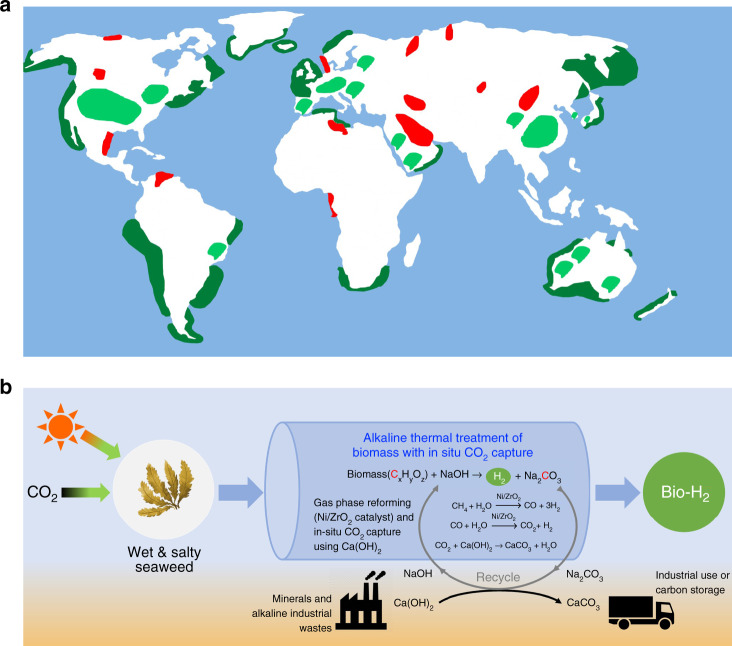
Distributed unconventional biomass resources and a reaction pathway to bio-hydrogen with carbon capture. **a** Global distribution of seaweed (green) and algae (light green) compared to major oil reserves (red). **b** Alkaline thermal treatment (ATT) of seaweed innovatively produces high purity bio-H_2_ from wet and salty biomass feedstock with a carbon capture and storage potential. Seaweed absorbs CO_2_ from atmosphere and stores the solar energy through photosynthesis without needing fresh water. The catalytic ATT involves the conversion of seaweeds in the presence of hydroxide and gas phase reforming catalyst. The overall reaction leads to bio-H_2_ as the carbon-free energy carrier, while carbon in biomass is stored as solid carbonate.

Seaweed is among less investigated types of bioenergy sources that is available worldwide, compared to the global oil distribution (Fig. 1a)^11–13^. Seaweeds and salt-tolerant algae can also be farmed in regions where algal biomass is currently cultivated (light green areas in Fig. 1a) by using brine instead of freshwater. Seaweeds have a global production of 216.6 thousands metric tons per year and have mainly been used for human foods, cosmetics, fertilizers, and chemical feedstock^9,14^. Interestingly, compared to terrestrial biomass, seaweed has a high average CO_2_ sequestration rate (36.7 ton hectare^−1^ year^−1^, seven times greater than that of conventional lignocellulosic biomass), rapid growth rate (harvested up to six times per year even without fertilizer), and does not compete with land-based food crops if grown in the sea^15,16^.

A few papers have reported on the use of seaweed as an energy source. Most of these studies have mainly focused on the biological conversion of seaweed;^17–19^ only a limited number of studies have focused on their thermochemical conversion^10,20,21^. Thermochemical conversion pathways of biomass (e.g., gasification) are attractive because of rapid reaction kinetics but are challenged by the need for dry feedstock. Thus, seaweeds and algae that have a high-moisture content (80–90%), generally need to be dried before their conversion^22,23^. Recently, subcritical and supercritical water gasification has been studied for wet biomass, e.g., algae and waste, but challenges remain given the high energy consumption due to high reactor pressure, low fuel (e.g., biocrude, CH_4_, H_2_, etc) yield, and salt precipitation^24,25^. Thus, an effective energy conversion technology that can convert wet and salty biomass into efficient energy fuels and energy carriers (e.g., H_2_) with high purity with reduced environmental footprints continues to be desired.

Extensive studies^26,27^ have demonstrated that alkali metals and alkali earth metals can enhance gasification, including promoted gasification rate, increased char conversion, reduced soot and tar formation. Umerki et al^28^ found that K can suppress the polycyclic aromatic hydrocarbon formations and promoted light tar decomposition. However, these reactions often require high reactor temperatures and generate low yield H_2_ as well as large amount CO_2_.

In this study, an alkaline thermal treatment (ATT) reaction is investigated by converting brown seaweeds in the presence of hydroxide (i.e., NaOH) and a gas-reforming Ni/ZrO_2_ catalyst. This particular reaction is less studied but very interesting in terms of its moderate reaction conditions (i.e., ambient pressure and temperature < 500 °C) that would allow the development of distributed biomass conversion systems without the need of a skilled operator. As shown in Fig. 1b, the overall ATT reaction is designed to push all the energy towards the H_2_ product, while the carbon in the seaweed is captured and stored as solid carbonates. If the purity of produced H_2_ is high enough to eliminate any subsequent gas cleaning steps, the overall biomass conversion technology would have a great potential to be sustainable. The biomass carbon captured in a form of solid carbonate can be stored with long-term stability in geologic formations, and if so, the overall ATT technology could achieve net carbon-negativity leading to a BioEnergy with Carbon Capture and Storage (BECCS) potential.

## Results

### H_2_ production from seaweed via ATT

Seaweed can have a wide range of moisture and salt content^23,29^. As a result, a dry ash-free (daf) mass, *m*_1_, provided in Eq. (1) is generally used to fairly compare different studies.

1 $$m_1 = m_0 \times \left( {1 - a} \right) \times \left( {1 - b} \right)$$

where *m*_0_ is the mass of biomass, *a* is the moisture content, and *b* is the ash content in total solid (TS). Brown seaweed procured for this study also contains a high ash content of 28.3 wt.% in total solid with a low moisture content of 7.8 wt.% (detailed composition is provided in Supplementary Table 1). In terms of carbon, brown seaweed is very rich in carbohydrates, mainly 10–40 wt.% alginates (C_6_H_8_O_6_), 2–34 wt.% laminarin (C_6_H_10_O_5_), 5–25 wt.% mannitol (C_6_H_14_O_6_), and 5–20 wt.% fucoidan (C_7_H_14_O_7_S)^10^.

Some interesting studies have been reported on the gasification^24,29,30^ and supercritical water (SCW) reaction^31–33^ of seaweed to enhance H_2_ production. SCW has been suggested as an effective means for producing H_2_ due to the ability of dissociated water to behave as an organic solvent^31^. The concept of using a base in the ATT reaction scheme can be analogous to the role of the OH^−^ ion from dissociated water in SCW^34,35^. Recent studies have demonstrated that the presence of NaOH in the ATT reaction greatly enhances H_2_ production from model biomass compounds such as glucose and cellulose under moderate reaction conditions (<500 °C) while capturing carbon in the form of solid carbonates^34–40^. The main differences between ATT and SCW/steam gasification include its relatively low reaction temperature and pressure, as well as a tolerance to salt and moisture.

The overall ATT reaction is described as follows with an example of cellulose:

2 $${\mathrm{C}}_6{\mathrm{H}}_{10}{\mathrm{O}}_5 + 12{\mathrm{NaOH}} + {\mathrm{H}}_2{\mathrm{O}} \to 6{\mathrm{Na}}_2{\mathrm{CO}}_3 + 12{\mathrm{H}}_2$$

In this reaction, NaOH participates in the fragmentation of cellulose and captures the produced CO_2_, thus generating high-purity H_2_ that can be directly used in various energy conversion systems such as a fuel cell without additional clean-up steps^36,41^. Since the ATT reaction involves water and salts, it inherently has a great potential to treat and utilize unconventional biomass with high salt and water content. However, ATT has not been demonstrated yet using a real biomass, particularly with seaweed. In this study, the ATT of brown seaweed is investigated and exemplified in the following ATT reactions:

3 $${\mathrm{C}}_6{\mathrm{H}}_8{\mathrm{O}}_6 + 12{\mathrm{NaOH}} \to 6{\mathrm{Na}}_2{\mathrm{CO}}_3 + 10{\mathrm{H}}_2\left( {{\mathrm{alginate}}\,{\mathrm{ATT}}} \right)$$

4 $${\mathrm{C}}_6{\mathrm{H}}_{10}{\mathrm{O}}_5 	+ 12{\mathrm{NaOH}} + {\mathrm{H}}_2{\mathrm{O}} \to 6{\mathrm{Na}}_2{\mathrm{CO}}_3 \\ 	+ 12{\mathrm{H}}_2\left( {{\mathrm{laminarin}}\,{\mathrm{ATT}}} \right)$$

5 $${\mathrm{C}}_6{\mathrm{H}}_{14}{\mathrm{O}}_6 + 12{\mathrm{NaOH}} \to 6{\mathrm{Na}}_2{\mathrm{CO}}_3 + 13{\mathrm{H}}_2\,\left( {{\mathrm{mannitol}}\,{\mathrm{ATT}}} \right)$$

6 $${\mathrm{C}}_7{\mathrm{H}}_{14}{\mathrm{O}}_7{\mathrm{S}} 	+ 16{\mathrm{NaOH}} + 2{\mathrm{H}}_2{\mathrm{O}} \to 7{\mathrm{Na}}_2{\mathrm{CO}}_3 \\ 	+ {\mathrm{Na}}_2{\mathrm{SO}}_4 + 17{\mathrm{H}}_2\left( {{\mathrm{fucoidan}}\,{\mathrm{ATT}}} \right)$$

**Fig. 2:**
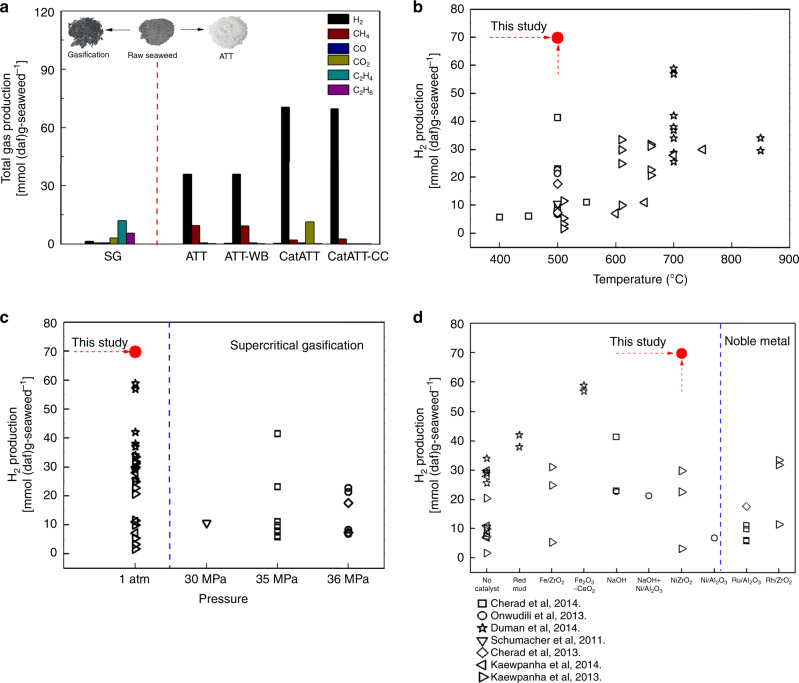
H_2_ production from brown seaweed via ATT and comparisons to previous seaweed gasification studies. **a** Major gaseous products generated via SG and ATT reactions performed at 500 °C and 1 atm under a steam atmosphere (temperature program given in the method section). The result is normalized to mmol/(daf)g-seaweed for the direct comparison to the previous seaweed conversion studies. **b–d** H_2_ production via CatATT-CC shown in **a** is compared to previous studies in terms of **b**—reaction temperature, **c**—reaction pressure and **d**—catalyst type.

The distinct conversion reaction of seaweed ATT is visibly shown in Fig. 2(a) (inset photos). The solid residue of steam gasification (SG) is as expected black char, whereas the solid product of the seaweed ATT reaction is a white powder illustrating the in situ fixation of biomass carbon in a solid carbonate form (white powder). In terms of H_2_ production, steam gasification generates a very low level of H_2_, and high levels of CO_2_ and many C_2_ hydrocarbon gases, indicating that it would be difficult to convert seaweed to pure H_2_ at low temperatures (e.g., 500 °C) via steam gasification. On the other hand, the ATT reaction results in a dramatically increased H_2_ production by 2573%. A small quantity of CH_4_ is also formed while both the evolution of C_2_ hydrocarbons and CO_2_ are suppressed. While sun-dried seaweeds are used for this study due to the ease of procurement and lab storage, the effect of water, supplied together with seaweed rather than from outside, on the ATT reaction is also investigated by pre-soaking seaweed to mimic real wet seaweed feedstock with 90% moisture content. The hydrated seaweed (ATT-WB) and dry seaweed (ATT) show very similar H_2_ formation behaviors, indicating that the ATT reaction can directly use both wet and dry seaweed feedstocks depending on how seaweed is harvested and transported. Once developed, the ATT reaction will be performed in a continuous mode feeding seaweed directly into a heated reactor system. Thus, any moisture from wet seaweed will quickly turn into steam and participate in the ATT reaction, for example as shown in Reaction (4) and (6).

Our prior studies on model biomass (e.g., cellulose) have shown that the Ni-based catalyst can be used to crack and reform minor products (e.g., light gases (CH_4_, C_2_H_4_, C_2_H_6_, etc) and condensable gases) to produce additional H_2_^36,37^. The overall H_2_ production doubles (CatATT), while the CO_2_ emissions also significantly increases, suggesting catalytic steam reforming reactions. To capture secondary CO_2_, Ca(OH)_2_ is used (CatATT-CC (carbon capture)) at the end of the ATT reaction. The Ni-based catalyst is shown to be stable and can be continuously reused (data given in Supplementary Fig. 1). The reactor configuration describing the overall reaction sequence is given in Supplementary Fig. 2.

Compared to the previously reported thermochemical conversion of seaweed^24,29–33,42^, the present study truly stands out. Cat-ATT-CC has achieved a high-purity H_2_ production of 69.69 mmol (daf)g-seaweed^-1^, which is considerably greater than those reported in the literature (Fig. 2b–d). While other studies often needed high reaction temperature and pressure, and/or a noble metal catalyst, the current ATT reaction can operate at a relatively low temperature (<500 °C) and under ambient pressure using a recyclable Ni catalyst. More importantly, the ATT of biomass can be applied to wet and salty feedstock (e.g., seaweed, food waste, algae) without any pretreatment, while traditional thermochemical biomass conversion processes require an energy-intensive pretreatment of a drying step^2,43,44^. Therefore, this catalytic ATT reaction combining high-purity H_2_ production with in situ CO_2_ capture in a single step process under moderate reaction conditions (<500 °C, 1 atm) has a great potential to be a transformative approach to unconventional biomass conversion.

### Carbon capture and storage potential of seaweed ATT

With ever-increasing concerns on climate change, the need for negative emission technologies has been widely argued^45–47^. There are only a few options that can achieve negative emission goals and BECCS is one of them. A dedicated carbon capture unit can be installed downstream on most of the traditional thermochemical biomass conversion system including those listed in Fig. 2b–d. However, a truly transformative technology would be an approach that can directly integrate biomass conversion with carbon capture.

**Fig. 3:**
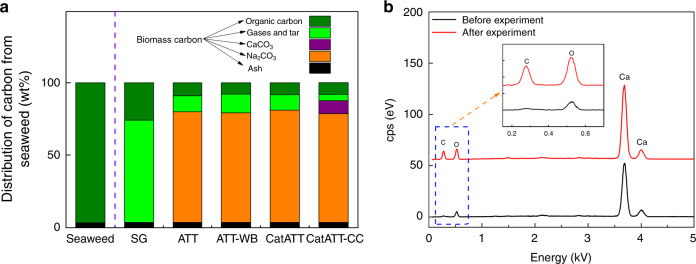
Fixation of seaweed carbon into solid carbonates. **a** Distribution of carbon from seaweed in different product streams illustrating the fate of carbon during the ATT reaction. Thermodynamically stable solid carbonates (red and orange) make up the most of the carbon fraction for CatATT-CC. **b** SEM-EDS of Ca(OH)_2_ before and after the CatATT-CC reaction confirming in situ carbon capture.

To elucidate its CCS potential, the fate of seaweed carbon throughout the ATT reaction is explored. The distribution of carbon in gas, liquid and solid phases after the ATT reaction is shown in Fig. 3a and the composition of seaweed ash is listed in Supplementary Table 2. The carbon in procured seaweed is mostly organic carbon with trace amounts of inorganic carbon later found in seaweed ash. As expected, steam gasification converts the organic carbon into gases and tars as well as char, and only a small portion (~3%) of carbon is found as inorganic carbon, indicating a high carbon content in its main product stream. On the other hand, during the ATT of seaweed, the organic carbon in the seaweed is mostly converted to Na_2_CO_3_ (~80%) and significantly reduced amounts of carbon-containing gases and tars (~13%). These results indicate that ATT can in situ capture carbon from seaweed biomass as sodium carbonates, which is thermodynamically stable and chemically safe to handle. The carbon distributions between phases remain nearly the same for ATT cases with and without the Ni-catalyst since Ni-catalyst mainly enhances gas phase reforming leaving reformed carbon as CO_2_ in the gas phase. By adding the additional in situ CO_2_ capture using Ca(OH)_2_, the final gaseous product is nearly carbon-free and the majority of seaweed organic carbon is pushed towards solid carbonates (Na_2_CO_3_ and CaCO_3_). Ca(OH)_2_ is used as the secondary CO_2_ capture material due to its low cost but other CO_2_ capture materials can also be used. Figure 3b shows the formation of CaCO_3_ based on enhanced C and O spectra after CatATT-CC, confirming the formation of CaCO_3_ (CO_2_ + Ca(OH)_2_ → CaCO_3_ + H_2_O).

Another distinctive nature of seaweed as an energy source is a high ash content since the ash component of biomass can have a considerable effect on its conversion reactions. The ash composition of the seaweed used in this study is analyzed using the X-ray fluorescence (XRF) (Supplementary Table 2). It has a large amount of K, Na, Ca, and Mg, which account for 95.78% of the seaweed ash. This brown seaweed ash can be classified as a K type based on Vassilev’s chemical classification of biomass ash^48^. K type ashes can enhance the leaching behavior, low-temperature transformation, and emission of volatiles during thermochemical reactions of biomass^36,48^. Moreover, the alkali and alkaline earth species (e.g., K, Na, and Ca) can enhance the biomass conversion via altering surface active sites during catalysis^49,50^. They can also contribute to reduced tar formation, catalyzed tar decomposition, and hindered char formation^49,51^. In this study, ash does not significantly impact SG and ATT reactions.

The ATT reaction effectively fixes the seaweed organic carbon into solid carbonates directly preventing CO_2_ emission, while other biomass conversion processes often require an additional carbon capture unit. Therefore, the unique ATT reaction has a great potential of BECCS while directly utilizing wet and salty biomass to produce high-purity H_2_.

### ATT reaction pathways generating high-purity H_2_

**Fig. 4:**
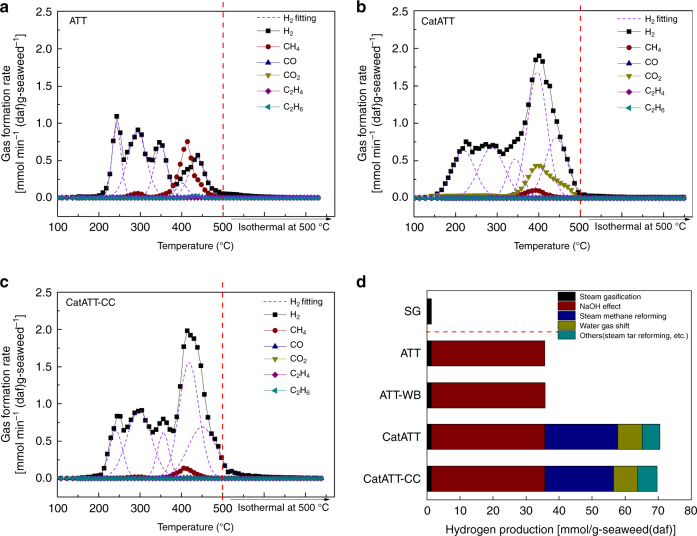
Gas formation behaviors of seaweed ATT and involved reactions. **a–c** Formation behaviors of major gaseous products during the ATT reaction of brown seaweed at various reaction temperatures. **a** ATT, **b** ATT with Ni-based catalyst, and **c** ATT with Ni-based catalyst and secondary CO_2_ capture using Ca(OH)_2_. **d** Pathways of H_2_ generation during the ATT reaction identified based on the results given in Fig. 2a.

To understand how the ATT reaction of seaweed combines high-purity H_2_ production with CO_2_ capture, gas formation behaviors are further investigated as reaction temperature is scanned from ambient to 500 °C. As shown in Fig. 4a, the non-catalytic ATT, H_2_ formation occurs throughout 150–500 °C while the CH_4_ formation is observed at a slightly higher temperature range (250–500 °C) with a major peak at 410 °C. The formation of CO and CO_2_ is insignificant.

When the Ni-catalyst is used downstream, the overall gas formation behaviors greatly change as seen in Fig. 4b. A large H_2_ formation peak at ~400–410 °C is detected with its formation rate reaching up to 2.0 mmol min^−1^(daf)g-seaweed^−1^. Within the same temperature range, both CH_4_ and CO_2_ concentrations are also greatly changed compared to the non-catalytic ATT case. The major peak of CH_4_ significantly decreases to below 0.2 mmol min^−1^(daf)g-seaweed^−1^, while CO_2_ formation greatly increases with the maximum 0.5 mmol min^−1^(daf)g-seaweed^−1^. The enhanced H_2_ and CO_2_ formation is clearly accompanied by the reduced CH_4_ formation. Therefore, gas reforming of CH_4_ and potentially light gases is the source of additional H_2_ formation observed in CatATT.

Ni-catalyst is well known for enhancing steam methane reforming (SMR) and water-gas shift (WGS)^34,35^.

7 $${\mathrm{CH}}_4 + {\mathrm{H}}_2{\mathrm{O}} \to {\mathrm{CO}} + 3{\mathrm{H}}_2$$

8 $${\mathrm{CO}} + {\mathrm{H}}_2{\mathrm{O}} \to {\mathrm{CO}}_2 + {\mathrm{H}}_2$$

From the results shown in Fig. 4a, b, 1 mol of CH_4_ generates ~4.19 mol of H_2_ and about 1.2 mol of CO_2_, which agree with the stoichiometries of reactions (7) and (8), in which 1 mol of CH_4_ generates 1 mol of CO and 3 mol of H_2_ via the SMR reaction; additionally, 1 mol of CO generates 1 mol of H_2_ and 1 mol of CO_2_ via the WGS reaction. Therefore, the secondary H_2_ formation is enhanced by the Ni-catalyst via the SMR and WGS reactions.

To capture the secondary CO_2_ generated during the WGS reaction and maintain the high-purity of H_2_, Ca(OH)_2_ was also placed in the system (CatATT-CC). As seen in Fig. 4c, the H_2_ formation behavior remains the same as the CatATT case, since the role of Ca(OH)_2_ is only CO_2_ capture. As designed, the CO_2_ peak disappears while maintaining the secondary H_2_ formation via SMR and WGS.

Based on these gas formation behaviors as well as the carbon distribution discussed earlier, it can be deduced that steam gasification given in reaction (6)^29^ is not significantly involved in H_2_ formation under temperatures below 500 °C.

9 $${\mathrm{seaweed}} + {\mathrm{H}}_2{\mathrm{O}} \to {\mathrm{H}}_2 + {\mathrm{CO}} + {\mathrm{CO}}_2 + {\mathrm{CH}}_4 + {\mathrm{CHs}} + {\mathrm{tar}} + {\mathrm{char}}$$

On the other hand, NaOH is directly involved in the ATT reaction of seaweeds and controls the gaseous intermediates and products as well as in situ capturing CO_2_. NaOH greatly improves the biomass conversion by 25 times leading to the H_2_ production of 35.69 mmol H_2_/(daf)g-seaweed compared to the low-temperature steam gasification case. The minor gaseous products containing carbon (e.g., CO, and CH_4_) are then reformed by the Ni/ZrO_2_ catalyst to produce up to 70.52 mmol-H_2_/(daf)g-seaweed (combined primary and secondary H_2_ formation) via the SMR and WGS reactions. In addition to light gases, tar can also contribute to the secondary H_2_ formation via catalytic steam tar reforming—reaction (10)^29,52^, although its contribution is not as significant (<9%) (Fig. 4d):

10 $${\mathrm{C}}_{\mathrm{n}}{\mathrm{H}}_{\mathrm{m}} + 2{\mathrm{n}}\,{\mathrm{H}}_2{\mathrm{O}} \to {\mathrm{n}}\,{\mathrm{CO}}_2 + \left( {2{\mathrm{n}} + {\mathrm{m}}/2} \right)\,{\mathrm{H}}_2$$

Ca(OH)_2_ effectively captures all CO_2_ during various secondary H_2_ formation reactions, and thus, the final gaseous product is high-purity H_2_. In summary, reactions (3–10) provide five pathways to H_2_ formation from seaweed biomass and their relative contributions are summarized in Fig. 4d.

### Regeneration of hydroxide via integrated mineral carbonation

NaOH exhibits an excellent performance in the ATT reaction of seaweed due to its high alkalinity and low melting point but the overall H_2_ production would not be net carbon-negative unless the produced carbonates can be safely stored for the long-term. CaCO_3_ produced from CO_2_ reaction with Ca(OH)_2_ can be permanently stored since it is insoluble and stable. Ca(OH)_2_ can be produced from alkaline industrial wastes and silicate minerals, and thus, it can be supplied with a low cost and a reduced carbon footprint^53^. On the other hand, Na_2_CO_3_ is highly soluble and NaOH is high in cost (USD921 per ton)^36^. Thus, in order to close the overall carbon loop and ultimately sequester seaweed carbon, it is highly desired to regenerate and re-use NaOH throughout the ATT reactions. The causticizing reaction (8) often used in the Kraft process of the pulp and paper industry can be employed to regenerate NaOH with Ca(OH)_2_ via the following metathesis reaction:

11 $${\mathrm{Na}}_2{\mathrm{CO}}_3 + {\mathrm{Ca}}\left( {{\mathrm{OH}}} \right)_2 \to {\mathrm{CaCO}}_3 + 2{\mathrm{NaOH}}$$

**Fig. 5:**
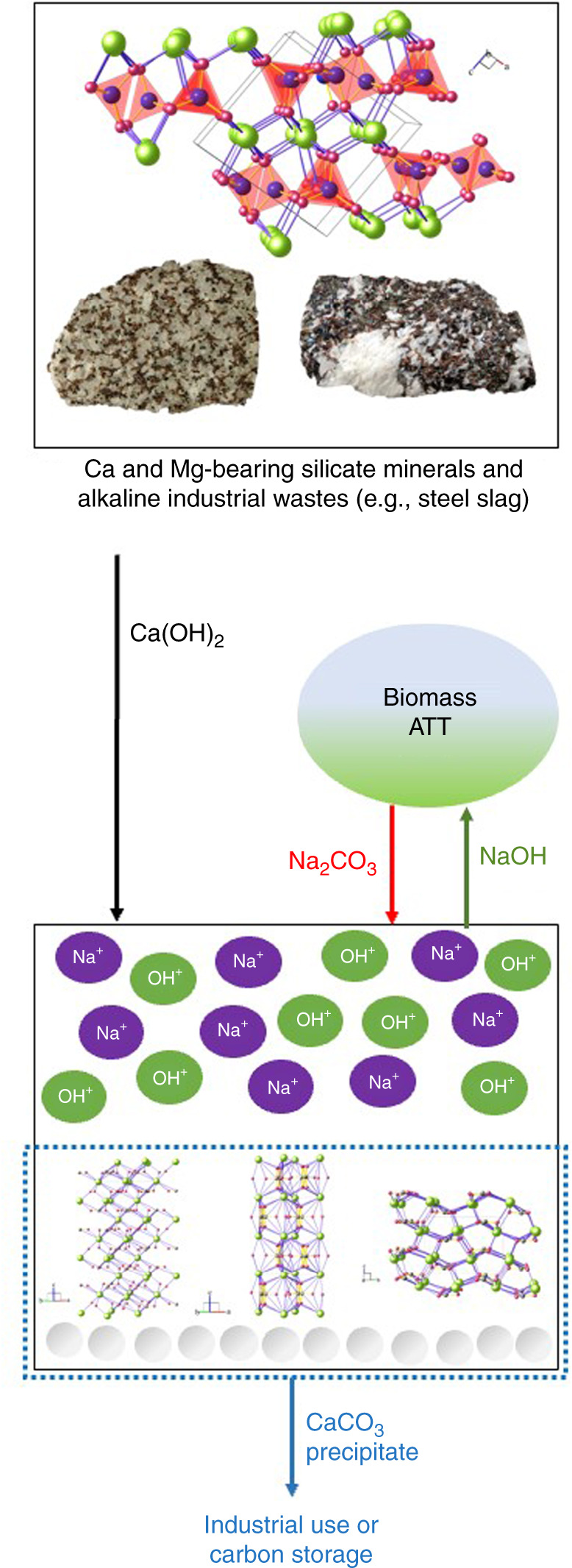
Regeneration of NaOH via metathesis reaction using Ca(OH)_2_ derived from minerals and alkaline industrial wastes. The ATT reaction produces high-purity H_2_ while fixing the seaweed carbon into solid carbonates (Na_2_CO_3_). The dry solid residue containing Na_2_CO_3_ can be regenerated on-site or easily transported to a centralized location for hydroxide regeneration. Ca(OH)_2_ from silicate minerals and alkaline industry wastes such as steel slag can be used to regenerate NaOH via a metathesis reaction. The regenerated NaOH is used for the subsequent ATT reaction cycle and the produced CaCO_3_ can be used as an industry product or stored to achieve the overall carbon negativity of the seaweed conversion technology.

As illustrated in Fig. 5, if the source of Ca(OH)_2_ is either alkaline industrial wastes (e.g., steel slag and waste concrete) or silicate minerals (e.g., wollastonite)^54–57^, the net carbon cycle could be neutral or even negative leading to a greater BECCS potential.

**Fig. 6:**
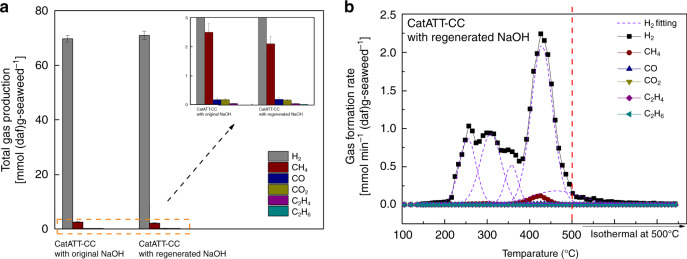
Recyclability of NaOH during the CatATT-CC reaction of brown seaweed. **a** Major gaseous products generated via CatATT-CC reactions performed using fresh NaOH and regenerated NaOH (at 500 °C and 1 atm under a steam atmosphere). **b** Gas formation behaviors in the CatATT-CC reaction of seaweed using regenerated NaOH.

The performance of the regenerated NaOH in the CatATT-CC reaction is investigated and Fig. 6a shows that the regenerated NaOH can attain similar H_2_ production as that of the fresh NaOH. Due to the solubility limitation of Ca(OH)_2_, the regenerated NaOH stream still contains significant amounts of Na_2_CO_3_ and trace amounts of Ca(OH)_2_ but the overall performance of NaOH is not impacted. Na_2_CO_3_ may accumulate throughout the ATT cycles, but it will not influence the overall reaction. As shown in the inset of Fig. 6a, CH_4_ formation and CO_2_ capture behaviors also remain the same for the regenerated NaOH. The comparison between gas formation behaviors shown in Figs. 4c and 6b also confirms that NaOH can be regenerated and reused for the ATT of seaweed producing high-purity H_2_.

From these data, a preliminary biomass-to-H_2_ conversion efficiency of the biomass ATT is estimated and compared with that of the conventional gasification and waster-gas shift reactions found in literature^58–60^. In order to provide similar system boundaries, Ca(OH)_2_ sorbent is used to capture CO_2_ from the water-gas shift reactor (details are provided in Supplementary Note 1). Results show that the biomass ATT can produce more H_2_ than the combined process of gasification and water-gas shift reactions. This is a very promising result considering a potential learning curve in the development of the biomass ATT technology, which is still in the early stage of development. Based on the findings from this mechanistic study, the biomass ATT can be further optimized in terms of the overall H_2_ production in an isothermal continuous reactor system, while improving the overall BECCS potential.

## Discussion

This study has shown that wet and salty seaweed can be directly used as a biomass feedstock to produce high-purity H_2_ without any pretreatment. The catalytic alkaline thermal treatment of brown seaweed has successfully produced 69.69 mmol H_2_/(daf)g-seaweed with in situ CO_2_ capture. The ATT reaction provides a considerably greater H_2_ production than those of other thermochemical methods under mild reaction conditions (<500 °C, 1 atm). NaOH can fragment the seaweed into relevant intermediates to increase the gaseous products including H_2_, while the Ni-based catalyst plays an important role in reforming gaseous intermediates to additional H_2_ via SMR and WGS reactions as well as tar reforming. The regeneration of NaOH and the stability of the Ni-based catalyst in the seaweed ATT reaction process show the effectiveness and feasibility of this innovative approach with a BECCS potential. The ATT of seaweed investigated in this study provides a novel pathway to utilize currently untapped unconventional biomass resources such as seaweed, food waste, and algae, which are often high in water and salt contents.

## Methods

### Biomass and reagents

Saccharina japonica, brown seaweed is obtained from Wando Island, South Korea and ground to <150 μm. While the proposed ATT reaction is developed for wet biomass, it would be difficult to store wet seaweed for long-term. Thus, the experiments are performed using readily-available sun-dried seaweed, which its salt content is retained in the biomass during the drying process (moisture content ~7.8%, salts content ~28%, by weight, respectively). NaOH and Ca(OH)_2_ are obtained from Sigma-Aldrich and used without further purification. The Ni/ZrO_2_ catalyst is prepared by dissolving 2752.6 mg of nickel(II) nitrate hexahydrate in 80 mL of ethanol. Then 5.0 g of ZrO_2_ powder (Alfa Aesar) is added to the solution. The mixture is stirred and heated to gradually impregnate the metal salt onto the ZrO_2_ support. The prepared catalyst is dried at 70 °C overnight and calcined in the air at 800 °C (heated at a ramping rate of 5 °C min^−1^) for 200 min. Finally, the catalysts are reduced under H_2_ atmosphere for 2 h at 500 °C. The prepared catalysts are analyzed using a NOVA 2200e for their specific surface area based on the Brunauer-Emmett-Teller (BET) method. The produced 10 wt.% Ni/ZrO_2_ catalyst has a specific surface area of 23.2 m^2^ g^−1^.

### Reactor system

The horizontal reactor consists of an inner quartz tube (2.54 cm in O.D. × 56.00 cm in length) and an outer three-zone split-tube furnace (Mellen Co., SC12R), as shown in Supplementary Fig. 3. The brown seaweed sample mixed with NaOH is placed in a ceramic boat in zone 1 and a thermocouple is installed to monitor the temperature during the ATT reaction. For the cases incorporating the catalyst and/or Ca(OH)_2_, they are separately placed in zone 2 of the fixed bed reactor to investigate their isolated effects in enhancing the formation of H_2_ and capture of CO_2_ generated from catalytic reaction. The reactor outlet is connected to a Micro GC (Inficon 3000) for the continuous analysis of gaseous products while collecting all the produced gas in a tedlar bag installed downstream for the analysis of minor products produced at very low concentrations.

### ATT reactions

Five distinct experimental combinations are designed to study the ATT reactions of brown seaweed and the roles of hydroxides and the catalyst (details given in Supplementary Table 3). In all cases, 1.0 g of mixed samples of a given molar ratio of brown seaweed and NaOH is loaded into a 10-mL ceramic boat, and placed in zone 1 of the horizontal reactor except for the steam gasification case performed without NaOH. The molar ratio of brown seaweed and NaOH is adjusted based on the reaction stoichiometry and all the results are normalized to dry ash-free (daf) grams of seaweed to enable accurate comparisons. For the experiments with catalysts and Ca(OH)_2_, 250.0 mg of 10 wt.% Ni/ZrO_2_ catalyst and (if used) 750.0 mg of Ca(OH)_2_ are separately placed in zone 2 of the fixed bed reactor. The carrier gas, N_2_, is introduced at 50 mL/min using a mass flow controller (Omega FMA5508) throughout the reaction. The reactor is first purged with N_2_ and then pre-heated to 100 °C (at a heating rate of 4 °C min^−1^) and maintained at that temperature for 30 min. After the pre-heating, water is injected into the hotbox at a rate of 0.023 mL min^−1^, where it is vaporized and carried by N_2_ to provide the steam for the ATT reaction. This creates a condition of an ATT reaction of wet biomass. While heating the reactor to 500 °C at a rate of 4 °C min^−1^, the H_2_ formation behavior of the ATT reaction of the brown seaweed is monitored. Subsequently, the reactor is maintained at 500 °C for 60 min before terminating each ATT experiment. The gaseous products from the reactor are passed through a condenser to separate the condensable compounds from light gases. The light gases are then analyzed online every 2.0 min using a Micro GC. The overall gas products are also collected in a tedlar bag for additional gas analysis.

### Gas analysis

Gas samples are analyzed online via a gas chromatography (GC) (Inficon Micro-GC 3000). The Micro GC has two 10-m Molsieve columns for H_2_, O_2_, N_2_, CH_4_, and CO analyses, and an 8 m Plot U for CO_2_ and C_2_H_6_ analyses. The detection limits are 20 ppm for H_2_, and 1ppm for O_2_, N_2_, CH_4_, CO, and CO_2_. N_2_ is used as a reference gas to quantify different gases and all the gas production was normalized to moles.

### Carbon analysis

The potential carbon-bearing product streams of the seaweed ATT reactions include carbon-containing gases (e.g., CO_2_, CO, CH_4_), liquid tar (C_2+_) and solid residue (organic and inorganic carbon (biochar and Na_2_CO_3_)). Both organic and inorganic contents of solid residues after the ATT reaction are determined using a UIC CM150 Coulometer with Total Carbon and Inorganic Carbon modules. The total carbon is calculated by combusting the residues in pure O_2_ at 900 °C, while the inorganic carbon is determined by dissolving the sample in perchloric acid. In both cases, the released amount of CO_2_ is measured to calculate the carbon content. The total carbon and inorganic carbon contents are used to determine the extent of the ATT reactions.

### Solid analysis

The ash composition of brown seaweed is analyzed using a PanAlytical Axios Advanced 4 kW WD XRF, which determines the elemental concentrations based on the X-ray intensity of each element. The detection limit of XRF is 100 ppm. The morphologies and elemental compositions of the solid samples before and after the ATT reactions are investigated using a Zeiss Sigma VP Scanning Electron Microscope with an Energy Dispersive Spectrometer (SEM-EDS). By evaluating the X-ray energies of the different elements, quantitative analysis is accomplished for each solid sample.

### Inductively coupled plasma optical emission spectrometer

The contents of Na and Ca cations in the solid samples obtained during the NaOH recycling step are analyzed using ICP-OES (ACTIVA-M, Horiba Jobin Yvon Inc., Edison, NJ). Based on the measured purity of the recycled hydroxide, its total mass added to the subsequent ATT reaction cycle is determined while maintaining the mass of NaOH constant.

## Supplementary information

Supplementary Information

Peer Review File

Supplementary Figure 1

Supplementary Figure 2

Supplementary Figure 3

## Data Availability

The authors declare that the main data supporting the findings of this study are available within the article and its Supplementary Information files. Extra data are available from the corresponding author upon request.
