# Supplementary material for: Alkaline thermal treatment of seaweed for high-purity hydrogen production with carbon capture and storage potential
Source: Nat Commun. 2020 Jul 29;11:3783. doi: 10.1038/s41467-020-17627-1 (PMC7391685; doi:10.1038/s41467-020-17627-1)
Supplement: Supplementary file 1 — Supplementary Information [file 41467_2020_17627_MOESM1_ESM.pdf]

Supplementary Information

Alkaline Thermal Treatment of Seaweed for High-purity Hydrogen Production with Carbon Capture and Storage Potential

Kang Zhang[1,2], Woo-Jae Kim[3,*] and Ah-Hyung Alissa Park[1,4,*]

[1]Lenfest Center for Sustainable Energy, Columbia University, New York, NY 10027, USA;
[2]State Grid Zhejiang Electric Power Research Institute, Hangzhou 310014, PR China;
[3]Department of Chemical Engineering and Materials Science, Ewha Womans University, Seoul 03760, Korea;
[4]Department of Earth and Environmental Engineering and Department of Chemical Engineering, Columbia University, New York, NY 10027, USA
*Correspondence
ap2622@columbia.edu (A.-H. A. Park)
wjkim1974@ewha.ac.kr (W.-J. Kim)

Supplementary Figures

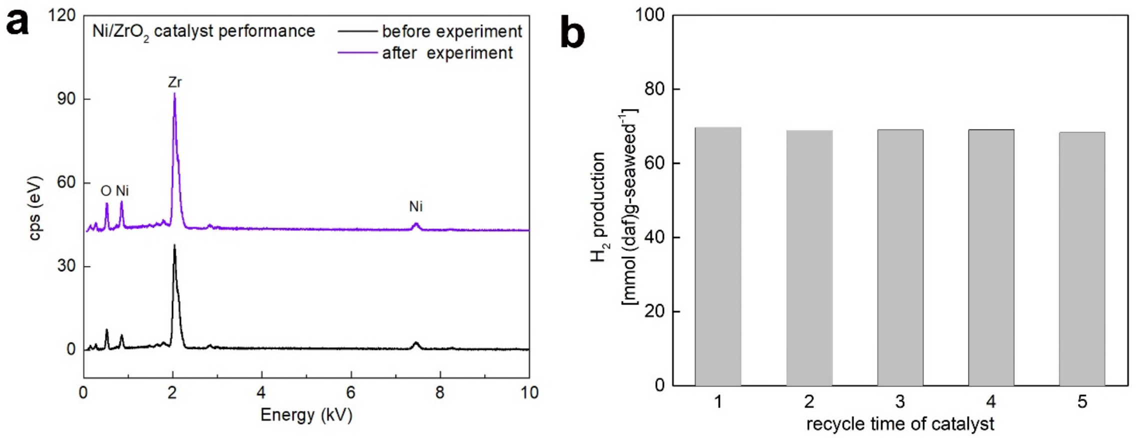

Supplementary Figure 1. Recyclability of the Ni-based catalyst in the CatATT-CC reaction. **a,** EDS spectra of the catalyst before and after the ATT reaction of seaweed. **b,** H_2 production from brown seaweed via CatATT-CC using recycled Ni-based catalyst (five cycles total).

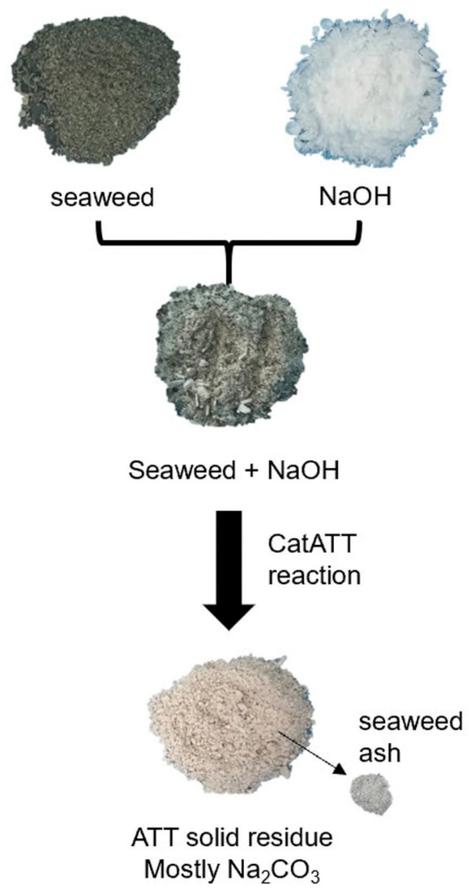

Supplementary Figure 2. CatATT-CC reaction of brown seaweed illustrated using the color of solid reactants and products. The mixture of seaweed (dark gray) and NaOH (white) was placed in zone 1 and produced the ATT gaseous intermediates and products. Compared to the initial color of seaweed, the residue from the seaweed ATT reaction was almost pure white confirming the fixation of seaweed carbon into solid carbonate, Na_2CO_3 (white). The residue showed slight gray tint due to the ash content. The separated ash (light gray) was also shown next to the photo of the ATT reaction residue.

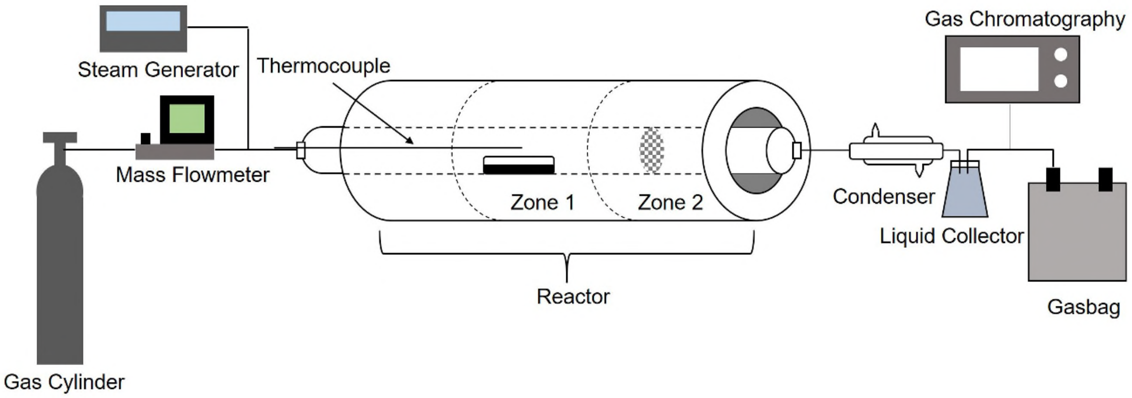

Supplementary Figure 3. Schematic of the reactor setup. The reactor consisted of an inner quartz tube (2.54 cm in O.D. × 56.00 cm in length) and an outer three-zone split-tube furnace (Mellen Co., SC12R). In all cases, mixed samples of a given molar ratio of brown seaweed and NaOH were loaded to a boat, and placed in zone 1 of the horizontal reactor except for the steam gasification case, which was performed without NaOH. For the experiments with catalysts and $Ca(OH)_2$, 10 wt.% Ni/ZrO_2 catalyst and/or $Ca(OH)_2$ were separately placed in zone 2 of the reactor. The ultimate design of the ATT reactor may unite all these components but in order to investigate the isolated effects and roles of hydroxide, catalyst and secondary hydroxide, two separate reaction zones were used in this study. The thermocouple was used to monitor the actual reaction temperature and a micro-GC (Inficon 3000) was used to analyze the gases online.

Supplementary Tables

Supplementary Table 1. Composition of brown seaweed

Components	*Saccharina japonica* (Brown seaweed)
Gravimetric analysis[a]	
Moisture Content (wt.%)	7.8
Total solids (TS) (wt.%)	92.2
Volatile solids (wt.% in TS)	71.7
Ash (wt.% in TS)	28.3
Elemental compositions in TS (dry mass)	
Carbon (wt.%)	31.5
Hydrogen (wt.%)	4.8
Oxygen (wt.%)	26.1
Nitrogen (wt.%)	1.5
Sulfur (wt.%)	0.6

[a]The seaweed sample was analyzed at 105 °C for 24 h and the volatile solid was analyzed at 550 °C for 8 hrs according to the commonly used protocol for seaweed analysis. All experiments were conducted in duplicates to ensure accuracy.

Supplementary Table 2. Ash composition of brown seaweed

Component[a]	Content (wt.% of ash)
SiO_2	0.66
Al_2O_3	0.25
Fe_2O_3	0.08
MgO	7.70
CaO	7.95
Na_2O	26.16
K_2O	53.97
TiO_2	n.d.
P_2O_5	3.23
MnO	n.d.
Cr_2O_3	n.d.
V_2O_5	n.d.

[a]The ash of brown seaweed was tested using a PanAlytical Axios Advanced 4kW WD XRF spectrometer. The detection limit was 100 ppm. n.d. = not detected

Supplementary Table 3. Experimental conditions

Reaction[a]	Zone 1		Zone 2	
	Seaweed[b]	NaOH[c]	Catalyst[d]	$Ca(OH)_2$[c]
Steam gasification (SG)	√			
Alkaline Thermal Treatment (ATT)	√	√		
ATT with wet biomass (ATT-WB) without steam	√	√		
Catalytic ATT (CatATT)	√	√	√	
CatATT with CO_2 capture (CatATT-CC)	√	√	√	√

[a]All the reactions were conducted in two stages: temperature ramping from 100 °C to 500 °C at 4 K/min followed by isothermal reaction at 500 °C for one hour. The reactor was maintained at 1 atm under steam atmosphere. [b]Seaweeds used in this study were Saccharina japonica procured from Wando Island, South Korea, and were ground to < 150 μm. [c]Chemicals were purchased from Sigma-Aldrich and used without further purification. [d]10 wt.% Ni/ZrO_2 catalyst was prepared using the impregnation method.

Supplementary Notes

Supplementary Note 1. Comparison of Biomass-to-H_2 Conversion Efficiencies

Based on the experimental data obtained in this study, a preliminary biomass-to-H_2 conversion efficiency of the biomass ATT is estimated and compared with that of the conventional gasification and waster-gas shift reactions found in literature.[1-3] In order to provide similar system boundaries for a better comparison, $Ca(OH)_2$ sorbent is used to capture CO_2 from the water-gas shift reactor.

Path 1. Partial oxidation and Water-Gas Shift reactions

(1) $C_{3.84}H_{6.13}O_{2.38}(s) + 1.32O_2(g) \rightarrow 2.645H_2(g) + 2.23CO(g) + 1.4CO_2(g) + 0.21CH_4(g)$[1]

$\Delta H = -320.03$ kJ/mol(1173K)

$\Delta H = -346.9$ kJ/mol (1453K)

(2) $CO(g) + H_2O(g) \rightarrow CO_2(g) + H_2(g)$ (CO conversion = 88%)[2]

$\Delta H = -49.6$ kJ/mol (593K)

- *Carbon capture* (95% of CO_2 is captured)[3]

(3) $Ca(OH)_2(s) + CO_2(g) \rightarrow CaCO_3(s) + H_2O(g)$ $\Delta H = -68.21$ kJ/mol (298K)[3]

Path 2. Alkaline thermal treatment

(4) $C_6H_{10}O_5(s) + 12NaOH(s) + H_2O(g) \rightarrow 6Na_2CO_3(s) + 12H_2(g)$ (η_4=0.71)

$\Delta H = -629.2$ kJ/mol (773K)

- NaOH regeneration (η_5=0.535, not optimized since it was not the focus of this particular study))

(5) $Ca(OH)_2(aq) + Na_2CO_3(aq) \rightarrow CaCO_3(s) + NaOH(aq)$ $\Delta H = +57.4$ kJ/mol (298K)

The data on the partial oxidation of seaweed was not available in the literature, and therefore, we used the data of other biomass (i.e., pine sawdust from Reference 1) for the H_2 production via partial oxidation (reaction (1)) for *Path 1*. They obtained 2.65 mol H_2 per 1 mol-biomass, from its partial oxidation.[1] Additional H_2 production was estimated using the WGS reaction (reaction (2)) using 1.1% Pt/CeO_2 catalyst (CO conversion of 88%) and 1.96 mol H_2 per 1 mol-biomass can be obtained.[2] Therefore, total of 4.61 mol H_2 / 1 mol-biomass can be obtained from *Path 1*.

On the other hand, our experimental data from this study showed that *Path 2* (biomass ATT) can produce 8.52 mol of H_2 / 1 mol-seaweed (reaction (4)). This is a very promising result

considering a future learning curve in the development of the biomass ATT technology. Based on the findings from this mechanistic and kinetic study, the biomass ATT can be further optimized in terms of the overall H_2 production in an isothermal continuous reactor system, while improving the overall Bio-Energy with Carbon Capture and Storage (BECCS) potential.

Supplementary References

1 Wang, Z. et al. Gasification of biomass with oxygen-enriched air in a pilot scale two-stage gasifier. Fuel 150, 386-393 (2015).

2 Hwang, K.-r., Ihm, S.-k. & Park, J.-s. Enhanced CeO_2-supported Pt catalyst for water–gas shift reaction. Fuel Processing Technology 91, 729-736 (2010).

3 Montes-Hernández, G., Renard, F., Geoffroy, N., Charlet, L. & Pironon, J. Calcite precipitation from CO_2–H_2O–Ca $(OH)_2$ slurry under high pressure of CO_2. Journal of Crystal Growth 308, 228-236 (2007).
