# Supplementary material for: Alkaline thermal treatment of seaweed for high-purity hydrogen production with carbon capture and storage potential
Source: Nat Commun. 2020 Jul 29;11:3783. doi: 10.1038/s41467-020-17627-1 (PMC7391685; doi:10.1038/s41467-020-17627-1)
Supplement: Supplementary file 2 — Peer Review File [file 41467_2020_17627_MOESM2_ESM.docx]

Peer Review Information

**Journal:** Nature Communications

**Manuscript title:** Alkaline Thermal Treatment of Seaweed for High-purity Hydrogen Production with Carbon Capture and Storage Potential

**Corresponding author name(s):** Prof Ah-Hyung Alissa Park

**Editorial notes:**

**none**

**Reviewer comments & decisions:**

**Reviewer comments, first version:**

Reviewer #1 (Remarks to the Author):

Review of Manuscript # NCOMMS-20-02208
Title: Alkaline Thermal Treatment of Seaweed for the Production of High-purity Hydrogen with Carbon Capture and Storage Potential
Authors: Kang Zhang, Woo-Jae Kim, and Ah-Hyung Alissa Park

This is a well designed new study. The manuscript is an important contribution to conversion of seaweeds for production of H2 with a novel reaction pathway to bio-hydrogen with carbon dioxide capture (CO2 sequestration). The alkaline thermal treatment (ATT) reaction of seaweeds can be described with a simplified overall reaction of cellulose as follows, where NaOH participates in the fragmentation of cellulose and captures the produced CO2 in the form of sodium carbonate, thus generating high-purity H2 (C6H10O5 + 12NaOH + H2O → 6Na2CO3 + 12H2) that can be directly used in various energy conversion systems such as a fuel cell without additional clean-up steps. Seaweeds are widely distributed unconventional salty and wet biomass resources which received little attention so far.

The data in Figure 4 shows that pyrolytic reactions do happen to form CH4, CO and CO2, but the use of Ni/ZrO2 catalyst significantly enhanced H2 production and suppressed CH4 and CO formation. When Ca(OH)2 was added, the CO2 product is captured and nearly pure H2 is produced. The catalytic ATT process has a great potential for further development of bio-energy because seaweeds growth does not require freshwater. The main operating costs of the new ATT process from this study are the use of sodium hydroxide and the thermal energy. Since most of the biomass conversion processes use thermal energy for conversion, such as pyrolysis, hydropyrolysis and gasification or reforming followed by water gas shift and gas separation. Thus this new process does not consume more energy than any existing thermochemical process but is much simpler and more energy efficient. Publication is recommended and the following questions should be addressed in the revised manuscript.

How is the sodium hydroxide involved in the H2 production reaction? Is the sodium hydroxide-enhanced H2 production due to kinetic or thermodynamic enhancement?

How do the alkali metal species such as Na, Ca and Mg affect the performance of the Ni/ZrO2 catalyst in continuous tests? The brown seaweeds contain these species and will likely impact the catalyst.

The authors reported the production of 69.69 mmol H2/(daf)gseaweed with in-situ CO2 capture. What is the theoretical maximum amount of H2 production using the brown seaweed?

What is the mass balance of the NaOH regeneration (Na2CO3 + Ca(OH)2 → CaCO3 + 2NaOH) ? In other words, how much NaOH is regenerated and how much Ca(OH)2 is reacted?


Reviewer #2 (Remarks to the Author):

This manuscript presents the work of the authors on the ATT of a high-salt Seaweed. The use of ATT to convert this type of feedstock to produce high-yield of hydrogen is interesting, especially considering the reaction conditions. However, some claims by the authors need to be validated or toned down. For instance, there are no data to support the claim for the process being potentially carbon-negative; authors would need to show evidence to support this. In addition, there is a general lack of statistical analysis throughout the manuscript; no evidence that the experiments were reproducible.
Other comments
Page 2, paragraph 2; there is need to emphasize that carbon-neutrality is only possible with sustainable biomass

Page 2 paragraph 3; there is need to make clear what the quantity of seaweed is on a global scale.
Page 9: the reaction equation is CO2 + Ca(OH)2 ==> CaCO3 + H2O
Page 12: I would suggest changing 2573% to a factor by which the parameter increased e.g. 20 times
Page 12; the section on regeneration of NaOH is a bit difficult to follow due to repetitions. Please check.
Could authors provide data in the manuscript about the amount of NaOH that could be recovered via recausticization?
Page 16; there are no evidence of kinetic parameters provided in this work; maybe just say 'mechanistic'
Page 18, the ninth line from the bottom; change 'creats' to 'creates'
Page 18: please state the assumptions related to the carbon analysis - it seems a combination of oxidation of organic carbon (char and fixed carbon) and thermal decomposition of inorganic carbon to obtain . There is no evidence that this method was validated by the authors.

**Author rebuttal, first version:**

**Responses to Reviewer #1’s comments**

This is a well-designed new study. The manuscript is an important contribution to conversion of seaweeds for production of H2 with a novel reaction pathway to bio-hydrogen with carbon dioxide capture (CO2 sequestration). The alkaline thermal treatment (ATT) reaction of seaweeds can be described with a simplified overall reaction of cellulose as follows, where NaOH participates in the fragmentation of cellulose and captures the produced CO2 in the form of sodium carbonate, thus generating high-purity H2 (C6H10O5 + 12NaOH + H2O → 6Na2CO3 + 12H2) that can be directly used in various energy conversion systems such as a fuel cell without additional clean-up steps. Seaweeds are widely distributed unconventional salty and wet biomass resources which received little attention so far.

The data in Figure 4 shows that pyrolytic reactions do happen to form CH4, CO and CO2, but the use of Ni/ZrO2 catalyst significantly enhanced H2 production and suppressed CH4 and CO formation. When Ca(OH)2 was added, the CO2 product is captured and nearly pure H2 is produced. The catalytic ATT process has a great potential for further development of bio-energy because seaweeds growth does not require freshwater. The main operating costs of the new ATT process from this study are the use of sodium hydroxide and the thermal energy. Since most of the biomass conversion processes use thermal energy for conversion, such as pyrolysis, hydropyrolysis and gasification or reforming followed by water gas shift and gas separation. Thus this new process does not consume more energy than any existing thermochemical process but is much simpler and more energy efficient. Publication is recommended and the following questions should be addressed in the revised manuscript.

First of all, we would like to thank the reviewer for all the positive comments, particularly acknowledging the novelty of our work. Here are the replies to the specific questions raised by Reviewer 1. The manuscript has also been revised accordingly.

Q1) How is the sodium hydroxide involved in the H2 production reaction? Is the sodium hydroxide-enhanced H2 production due to kinetic or thermodynamic enhancement?

**Reply:** The role of sodium hydroxide is multifaceted and discussed throughout the manuscript. For example, in the first part of the Result section, “*The concept of using a base in the ATT reaction scheme can be analogous to the role of the OH^-^ ion from dissociated water in SCW*.” “*In this reaction, NaOH participates in the fragmentation of cellulose and captures the produced CO2, thus generating high-purity H2 that can be directly used in various energy conversion systems such as a fuel cell without additional clean-up steps*.”

Sodium hydroxide has high alkalinity and low melting point, which can be dissociated into OH^-^ ion during the ATT reaction. It can fragment seaweed into relevant intermediates to increase the gaseous products including H2, which is thermodynamic enhancement. It also changes the ATT reaction rate via in-situ capturing the reaction product, CO2 leading to the kinetic enhancement. Therefore, our ATT reaction scheme integrated with carbon capture has both kinetic and thermodynamic enhancements to the H2 production.

Q2) How do the alkali metal species such as Na, Ca and Mg affect the performance of the Ni/ZrO2 catalyst in continuous tests? The brown seaweeds contain these species and will likely impact the catalyst.

1

**Reply:** This is a great question. Many of the conventional biomass conversion reactions can suffer from high salt contents leading to catalyst poisoning. Since our ATT reaction uses alkaline metal hydroxides (i.e., NaOH, Ca(OH)2, Mg(OH)2), these species in brown seaweeds do not hinder or impact our ATT reactions. Compared to the amounts of Na, Ca and Mg added as hydroxides for the ATT reaction, Na, Ca and Mg in seaweeds are minimal and they would behave similarly to the added Na, Ca and Mg. This is the reason that our technology is unique and can be applied to salty biomass such as seaweed.

Furthermore, the reaction temperature of ATT is lower than those of other thermochemical reactions (e.g., pyrolysis and gasification), and thus, Na, Ca and Mg compounds would stay in the solid phase (or thin liquid layer) during the reaction. Also, as shown in Figure S1 (supplementary information), the Ni/ZrO2 catalyst is placed in the downstream of the ATT reaction zone to promote the gas phase reforming: seaweed+NaOH (zone 1) followed by Ni catalyst+Ca(OH)2 (zone 2). Thus, there is no direct contract between alkali metal species (from both added hydroxide and seaweed) and catalyst.

The stability of the Ni/ZrO2 catalyst was evaluated and the data are given in Supplementary Figure S3. Alkali metal species did not impact the performance of catalytic ATT reaction and the Ni/ZrO2 catalyst could be reused as mentioned in lines 134-135, “*The Ni-based catalyst is shown to be stable and can be continuously reused*.”

Q3) The authors reported the production of 69.69 mmol H2/(daf)g seaweed with in-situ CO2 capture. What is the theoretical maximum amount of H2 production using the brown seaweed?

**Reply:** We calculated the theoretical maximum amount based on the following reaction and it was 98.33 mmol H2/(daf)g-seaweed. The chemical composition of brown seaweed (C2.625H4.8O1.63125N0.10714S0.01875) was derived from the element compositions of seaweed given in Supplementary Information.

C2.625H4.8O1.63125N0.10714S0.01875 + 5.39464 NaOH + 1.24553 H2O → 2.625 Na2CO3 + 0.10714 NaNO3 + 0.01875 Na2SO4 + 6.34285 H2

Using the theoretical maximum amount of H2 calculated from this ATT reaction of seaweed, the maximum biomass-to-H2 conversion obtained from this study was reported as 71%.

2

Q4) What is the mass balance of the NaOH regeneration (Na2CO3 + Ca(OH)2 → CaCO3 + 2NaOH) ? In other words, how much NaOH is regenerated and how much Ca(OH)2 is reacted?

**Reply:** Yes, that is the reaction for the hydroxide regeneration. For this reaction, we mixed stoichiometric amounts of Na2CO3 and Ca(OH)2 in the water and recovered (regenerated) 53.5% of original NaOH at room temperature. We believe that the reaction conditions (e.g., temperature and pH) can be further optimized to improve regeneration efficiency but it was not the focus of this study.

We have found a patent by Honghi[1] stating “The efficiency of the reaction of sodium carbonate with lime to regenerate caustic soda is limited by chemical equilibria and kinetics such that causticizing efficiency is typically in the range of about 78 to 83%. The method of this invention can increase the causticizing efficiency in an alkaline pulping process from about 81% up to about 89%.” This reaction (Na2CO3 + Ca(OH)2 → CaCO3 + 2NaOH) is a relatively common reaction (e.g., conventionally used in pulp and paper industry), and thus, it should not be a bottleneck for the biomass ATT technology. Our on-going study will provide additional data on the recovery of NaOH.

[1] Honghi N. Tran, Charles M. Bair, and Robert B. McBroom. "Method of increasing the causticizing efficiency of alkaline pulping liquor by borate addition." U.S. Patent No. 6348, 128. 19 Feb. 2002.

3

**Responses to Reviewer #2’s comments**

This manuscript presents the work of the authors on the ATT of a high-salt Seaweed. The use of ATT to convert this type of feedstock to produce high-yield of hydrogen is interesting, especially considering the reaction conditions. However, some claims by the authors need to be validated or toned down.

We thank the reviewer for the positive comment on the novelty of our research. The detailed comments were insightful and helpful in revising our manuscript. Here are thorough responses to each of reviewer’s questions and comments. The manuscript has been revised according to recommendation made by Reviewer #2.

Q1) For instance, there are no data to support the claim for the process being potentially carbon-negative; authors would need to show evidence to support this. In addition, there is a general lack of statistical analysis throughout the manuscript; no evidence that the experiments were reproducible.

**Reply:** We appreciate reviewer’s comments. As the reviewer pointed out, our ATT process itself starts as carbon-neutral (NOT carbon negative) like any other biomass conversion technologies. Our ‘carbon negative’ potential comes from the in-situ CO2 capture leading to the formation of solid carbonates (e.g., Na2CO3). By converting CO2 produced from seaweed into solid carbonates, we decarbonized the gaseous product (H2). The question becomes what to do with the produced solid carbonate. If we eventually release CO2 from the produced solid carbonate, the overall process become ‘carbon neutral’ from ‘carbon negative.’ That is the reason that the hydroxide regeneration step is very important. By producing insoluble CaCO3 while regenerating NaOH, the overall bioenergy process via ATT can be ‘carbon negative’ as long as Ca used in this reaction is not from fresh limestone. As discussed in the manuscript, the use of Ca from industrial wastes is considered to be beneficial since its carbon footprint can be considered neutral and the overall process will also address the issue of solid waste management issue. We added more discussion on this to the revised manuscript to further clarify.

To answer the reproducibility issue (statistical analysis) raised by the reviewer, we added the error bars to all Figures in the revised manuscript (e.g., see Figure 2a). We should have added these at the first place. Thank you for pointing out the missing error bars. As shown in Figure 2a, the ATT reaction was highly reproducible.

We also tested the recyclability of NaOH (shown in Figure 6a) and Ni/ZrO2 catalyst (Figure S3 in Supplementary Information) as discussed in the manuscript (Lines 134-135): “*The Ni-based catalyst is shown to be stable and can be continuously reused (data given in Supplementary Figure S3)*.”

4

**Figure S3.** Recyclability of the Ni-based catalyst in the CatATT-CC reaction. a) EDS spectra of the catalyst before and after the ATT reaction of seaweed. B) H2 production from brown seaweed via CatATT-CC using recycled Ni-based catalyst (five cycles total).

Q2) Page 2 paragraph 2; there is need to emphasize that carbon-neutrality is only possible with sustainable biomass.

**Reply:** We definitely agree with the reviewer on this point. Following your comment, we revised lines 25-31 to “*Not all biomass can be considered sustainable resources since their cultivation, harvesting, treatment, processing and transportation can lead to greater environmental impacts than benefits depending on the paths have been taken. For example, the transportation of low energy density biomass for long distance using fossil fuels would make biomass less attractive as sustainable resources. Nevertheless, biomass is one of the renewable resources that have a potential to be not only carbon-neutral but even be carbon-negative if combined with carbon capture and storage (CCS) scheme*.”

Q3) Page 2 paragraph 3; there is need to make clear what the quantity of seaweed is on a global scale.

**Reply:** Thank you for your comment. We added the global annual seaweed production with additional references in lines 41-43 of the revised manuscript, “*Seaweeds have a global production of 216.6 thousand metric tons per year and have mainly been used for human foods, cosmetics, fertilizers, and chemical feedstock*.”9,14

Q4) Page 9: the reaction equation is CO2 + Ca(OH)2 ==> CaCO3 + H2O

**Reply:** Thank you very much for pointing out the mistake. We corrected the reaction equation, “*Figure 3(b) shows the formation of CaCO3 based on enhanced C and O spectra after CatATT-CC, confirming the formation of CaCO3 (CO2 + Ca(OH)2 → CaCO3 + H2O)*.” (Lines 176-178)

Q5) Page 12: I would suggest changing 2573% to a factor by which the parameter increased e.g. 20 times

**Reply:** Following your suggestion, we changed ‘2573%’ to ‘25 times.’ (Line 232)

Q6) Page 12; the section on regeneration of NaOH is a bit difficult to follow due to repetitions. 5

Please check.

**Reply:** As suggested, we removed the repeated paragraph and revised the paragraph to “*CaCO3 produced from CO2 reaction with Ca(OH)2 can be permanently stored since it is insoluble and stable. Ca(OH)2 can be produced from alkaline industrial wastes and silicate minerals, and thus, it can be supplied with a low cost and a reduced carbon footprint. On the other hand, Na2CO3 is highly soluble and NaOH is high in cost (USD921 per ton).^38^*” (Lines 247-250)

Q7) Could authors provide data in the manuscript about the amount of NaOH that could be recovered via recausticization?

**Reply:** We provided the NaOH regeneration data to the section “Comparison of Biomass-to-H2 Conversion Efficiencies” in the Supplementary Information. For this reaction, we mixed stoichiometric amounts of Na2CO3 and Ca(OH)2 in the water and recovered (regenerated) 53.5% of original NaOH at room temperature. We believe that the reaction conditions (e.g., temperature and pH) can be further optimized to improve regeneration efficiency but it was not the focus of this study.

We have found a patent by Honghi[1] stating “The efficiency of the reaction of sodium carbonate with lime to regenerate caustic soda is limited by chemical equilibria and kinetics such that causticizing efficiency is typically in the range of about 78 to 83%. The method of this invention can increase the causticizing efficiency in an alkaline pulping process from about 81% up to about 89%.” This reaction (Na2CO3 + Ca(OH)2 → CaCO3 + 2NaOH) is a relatively common reaction (e.g., conventionally used in pulp and paper industry), and thus, it should not be a bottleneck for the biomass ATT technology. Our on-going study will provide additional data on the recovery of NaOH.

[1] Honghi N. Tran, Charles M. Bair, and Robert B. McBroom. "Method of increasing the causticizing efficiency of alkaline pulping liquor by borate addition." U.S. Patent No. 6348, 128. 19 Feb. 2002.

Q8) Page 16; there are no evidence of kinetic parameters provided in this work; maybe just say 'mechanistic'

**Reply:** We agree with the reviewer’s comment. We changed the sentence to “*Based on the findings from this* ***mechanistic*** *study, the biomass ATT can be further optimized in terms of the overall H2 production in an isothermal continuous reactor system, while improving the overall Bio-Energy with Carbon Capture and Storage (BECCS) potential.”*

Q9) Page 18, the ninth line from the bottom; change 'creats' to 'creates'
    **Reply:** Thank you for pointing out the typo. It has been fixed.
Q0) Page 18: please state the assumptions related to the carbon analysis - it seems a combination of oxidation of organic carbon (char and fixed carbon) and thermal decomposition of inorganic carbon to obtain. There is no evidence that this method was validated by the authors.

**Reply:** We have added the following statements to the revised manuscript to further clarify the

6

carbon that can be found in the reaction product streams. “*The potential carbon-bearing product streams of the seaweed ATT reactions include carbon-containing gases (e.g., CO2, CO, CH4), liquid tar (C2+) and solid residue (organic and inorganic carbon (biochar and Na2CO3))*.” We have also discussed this point in the annotation of Figure 3 - “*Distribution of carbon from seaweed in different product streams illustrating the fate of carbon during the ATT reaction. Thermodynamically stable solid carbonates (red and orange) make up the most of the carbon fraction for CatATT-CC*.”

**Reviewer comments, second version:**

Reviewer #1 (Remarks to the Author):

The authors have properly addressed the comments and questions this reviewer raised on their original manuscript. This is really a nice work and I recommend the publication in Nature Comm.


Reviewer #2 (Remarks to the Author):

The revision is well done but the claim that the reaction pathway is novel should be reconsidered, in the light of the following publications;

Hui Zhou, Ah-Hyung Alissa Park, Bio-energy with carbon capture and storage via alkaline thermal Treatment: Production of high purity H2 from wet wheat straw grass with CO2 capture. Applied Energy 264 (2020) 114675.

Ishida et al., One-step production of CO- and CO2-free hydrogen from biomass, J Chem Technol Biotechnol 80:281–284 (2005), DOI: 10.1002/jctb.1188

**Author rebuttal, second version:**

**Responses to Reviewer #1’s comments**

The authors have properly addressed the comments and questions this reviewer raised on their original manuscript. This is really a nice work and I recommend the publication in Nature Communications.

Thank you for your very positive comments. We anticipate that our finding will significantly contribute to the field of BioEnergy with Carbon Capture and Storage (BECCS).

1

**Responses to Reviewer #2’s comments**

The revision is well done but the claim that the reaction pathway is novel should be reconsidered, in the light of the following publications;

Hui Zhou, Ah-Hyung Alissa Park, Bio-energy with carbon capture and storage via alkaline thermal Treatment: Production of high purity H2 from wet wheat straw grass with CO2 capture. Applied Energy 264 (2020) 114675.

Ishida et al., One-step production of CO- and CO2-free hydrogen from biomass, J Chem Technol Biotechnol 80:281–284 (2005), DOI: 10.1002/jctb.1188

Thank you for the reviewer’s comments. As the reviewer pointed out, the reaction pathway itself (alkaline thermal treatment reaction between biomass and hydroxide to produce hydrogen) has been investigated in the past – mostly led by my research group since 2009. It is great that you have found one of our latest papers published two months ago (Zhou and Park, 2020).

As we discussed in our previous papers, the inspiration of the alkaline thermal treatment of biomass came from the work by Ishida et al (2005). They published two key papers suggesting the potential benefits of hydrogen production via ATT reaction. However, their work was limited in terms of mechanistic studies and feedstock variability. They claimed that biomass ATT can produce CO-free H2 which was not always true. Thus, our research since 2009 has been an interesting journey to fundamentally investigate how ATT reaction can produce H2 while suppressing COx formation. Further, Ishida et al did not discuss how to regenerate hydroxide to complete the cycle. They have not pursued this research since then.

It was our group that has found a unique opportunity of this particular reaction for the BECCS potential. My paper (Zhou and Park, 2020) focuses on the ATT reaction of land-based biomass, which consists of typical sugars including cellulose and they are not wet or salty.

While our current work is based on a similar ATT reaction scheme, this is the first study on real seaweed, whose carbohydrates are very different from grass grown in land. As discussed in the manuscript, the use of seaweed as the resource offers an important unconventional opportunity for bioenergy. Unlike land-based biomass, seaweeds have high water and salt contents, and thus, many conventional biomass conversion technologies would not work. Here we showed that ATT of seaweed innovatively produces high-purity bio-H2 from wet and salty biomass feedstock with a carbon capture and storage potential.

Since we already emphasized the novelty of our study in the manuscript, we deleted the phrase "novel" in our revised manuscript.

**Reviewer comments, third version:**

**Author rebuttal, third version:**
